# Please Place Your Seat in the Full Upright Position: A Technical Framework for Landing Upright Radiation Therapy in the 21^st^ Century

**DOI:** 10.3389/fonc.2022.821887

**Published:** 2022-03-03

**Authors:** Sarah Hegarty, Nicholas Hardcastle, James Korte, Tomas Kron, Sarah Everitt, Sulman Rahim, Fiona Hegi-Johnson, Rick Franich

**Affiliations:** ^1^School of Science, RMIT University, Melbourne, VIC, Australia; ^2^Department of Physical Sciences, Peter MacCallum Cancer Centre, Melbourne, VIC, Australia; ^3^Centre for Medical Radiation Physics, University of Wollongong, Wollongong, NSW, Australia; ^4^Sir Peter MacCallum Department of Oncology, Faculty of Medicine, Dentistry and Health Science, University of Melbourne, Parkville, VIC, Australia; ^5^Department of Biomedical Engineering, School of Engineering, University of Melbourne, Melbourne, VIC, Australia; ^6^Department of Radiation Therapy, Peter MacCallum Cancer Centre, Melbourne, VIC, Australia; ^7^Department of Radiation Oncology, Peter MacCallum Cancer Centre, Melbourne, VIC, Australia

**Keywords:** patient positioning, upright, radiation therapy, lung cancer, immobililization

## Abstract

Delivering radiotherapy to patients in an upright position can allow for increased patient comfort, reduction in normal tissue irradiation, or reduction of machine size and complexity. This paper gives an overview of the requirements for the delivery of contemporary arc and modulated radiation therapy to upright patients. We explore i) patient positioning and immobilization, ii) simulation imaging, iii) treatment planning and iv) online setup and image guidance. Treatment chairs have been designed to reproducibly position seated patients for treatment and can be augmented by several existing immobilisation systems or promising emerging technologies such as soft robotics. There are few solutions for acquiring CT images for upright patients, however, cone beam computed tomography (CBCT) scans of upright patients can be produced using the imaging capabilities of standard Linacs combined with an additional patient rotation device. While these images will require corrections to make them appropriate for treatment planning, several methods indicate the viability of this approach. Treatment planning is largely unchanged apart from translating gantry rotation to patient rotation, allowing for a fixed beam with a patient rotating relative to it. Rotation can be provided by a turntable during treatment delivery. Imaging the patient with the same machinery as used in treatment could be advantageous for online plan adaption. While the current focus is using clinical linacs in existing facilities, developments in this area could also extend to lower-cost and mobile linacs and heavy ion therapy.

## Introduction

Radiation therapy is delivered predominantly to patients in the recumbent position on a treatment couch, with a gantry rotating around them to deliver radiation from prescribed angles. Recumbent positioning is intrinsically linked to the acquisition of the required volumetric imaging used for treatment planning. The inherent stability this position provides was particularly important for early computed tomography (CT) scanners, which were quite slow (1).

There are several potential advantages to treating selected patients in an upright position instead of recumbent (2). Some conditions, such as obesity, heart problems, superior vena cava obstruction and phrenic nerve injury, can result in respiratory difficulty when in a supine position resulting in uncomfortable treatment (3, 4). Patients may be unable to complete treatment as a result or have compromised treatment due to poor position stability. Patient position can also influence the position of some tumours and organs at risk (OARs). Treating mediastinal tumours in upright patients resulted in reduced normal lung tissue irradiation due to an increase in lung volume (5–7). An upright position can also reduce the effect of respiratory motion, potentially resulting in reduced normal tissue dose (8). Further, with appropriate immobilization, an upright patient could be comfortably rotated relative to a fixed beam to vary the treatment angle, reducing the need for a rotating gantry (9). Gantry-free treatment is being explored *via* horizontal patient rotation but is complicated by challenges such as angle-dependent patient deformation due to gravity (10, 11). Importantly, the vertical rotation could be done faster than a gantry rotation around the patient (increasing from less than 1 rpm to as much as 3-7 rpm, such as used for Total Skin Electron Treatment) thereby increasing scope for breath-hold and improving image quality (12, 13).

The present work is focused on implementing upright positioning for routine, contemporary photon treatments, including intensity and field-modulated capabilities, using clinical linear accelerators. Aside from the immediate potential benefits outlined above, upright patient positioning may facilitate shrinkage of the machine and shielding and reduction in machine complexity. Such size and complexity reductions open up the potential for portable and low-physical footprint radiation therapy devices. However, other modalities may likely benefit from further addressing the challenges associated with upright radiation therapy. Indeed, some work in this direction already exists in the context of particle beam therapies (14). A dedicated system exists to deliver particle therapy to upright patients *via* a suite of specifically designed equipment (15). While comprehensive, the system is only likely to be implemented in the largest or most specialised centres. The high cost and space requirements of rotating gantries, especially for very heavy ions such as carbon, make upright patient geometry attractive in this setting.

We previously reviewed the historical applications of upright radiation therapy, relevant recent developments and potential benefits (2). While upright radiation therapy has been delivered in various forms, these have typically been limited in complexity and often for palliative intent. The current paper describes the technical requirements and potential solutions for the delivery of radiation therapy to upright patients of the same quality as that achieved in current recumbent treatments. Our current focus relates to state-of-the-art delivery *via* implementation in existing linear accelerator facilities, with a minimum of bespoke additional equipment. This is likely to produce the most immediate clinical benefits and allow for upright patient positioning to become a routine treatment modality option. The current framework covers patient positioning and immobilization, simulation imaging, treatment planning and online setup and image guidance (see **Table 1**).

**Table 1:** Overview of the key requirements that need to be met for clinical upright radiation therapy, and the proposed solutions that are detailed in this paper. This includes patient position and immobilization, treatment planning imaging, treatment planning, and online setup and image guidance.

	Requirements	Proposed solution
**Patient positioning and immobilization**		
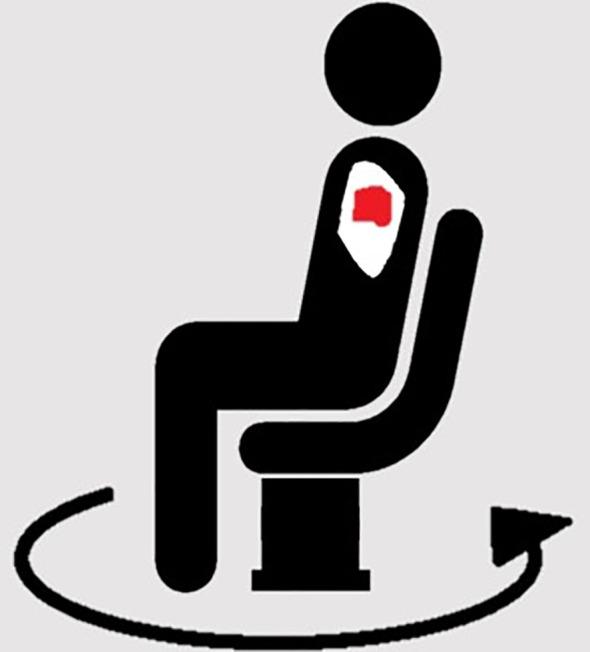	• Upright, reproducible, stable, comfortable position • Rotate patient relative to the treatment beam • Angular control with feedback to the delivery system	• Chairs and standing frames • Adapt existing immobilization devices • Augmented by soft robotics • Turntable with interfaced control
**Treatment planning imaging**		
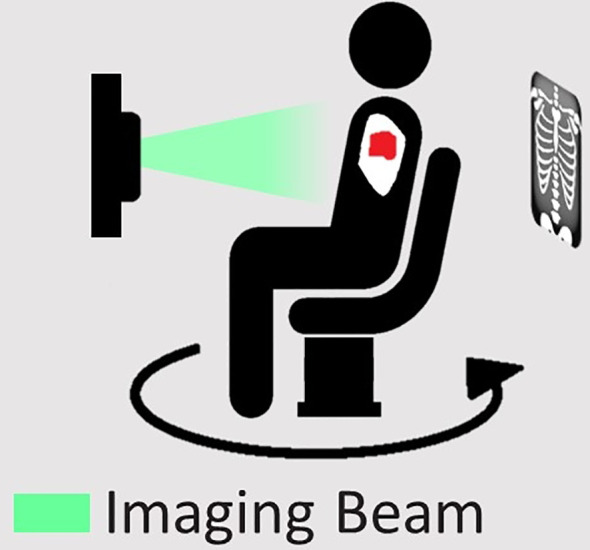	• High-quality 3D and 4D images of an upright patient • Accurate tissue classification/HU for dose calculation • Geometrically accurate	• Upright CT/MRI scanner • Linac onboard CBCT with HU corrections • Deformable registration from recumbent multi-modality imaging
**Treatment planning**		
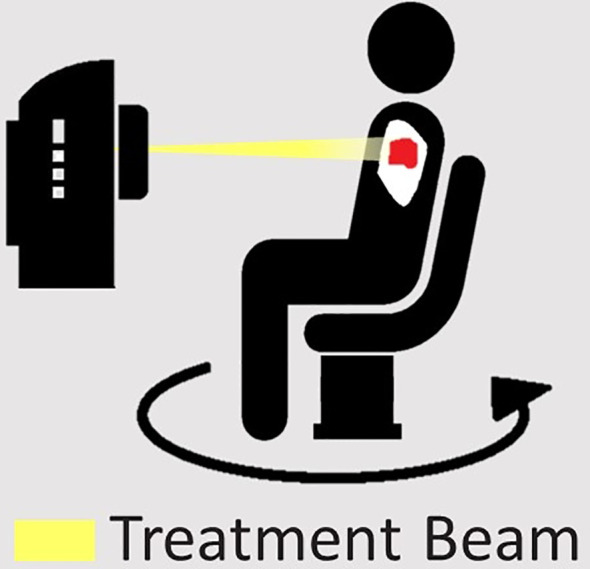	• TPS accepts upright treatment geometry • Gantry rotation transposed to patient rotation • Implementation of 3DCRT, IMRT, VMAT	• Expand upright geometry availability to photon/electrons • Turntable rotation included as planning and optimization variable
**Online setup and image guidance**		
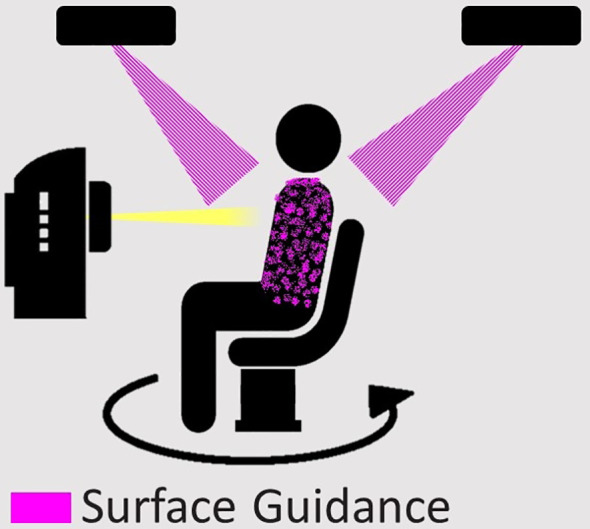	• In-room patient setup: pose and isocenter alignment • Planar and volumetric imaging for tumour targeting • Continuous patient position monitoring	• Laser/surface guidance systems adapted for an upright position • Upright 2D/3D/4D on-board imaging using kV/MV photons or MRI • Online position monitoring adapted for a continuously moving patient

## Patient Positioning and Immobilization

Contemporary radiation therapy is delivered using beams from multiple angles, or continuous arcs around the patient, to improve conformity and reduce dose to critical organs. A fundamental requirement is thus rotation of the radiation beam around the patient, or rotation of a patient relative to a static beam. Transposition of the primary anatomical axis relative to the conventional treatment orientation and the gantry rotation axis will be required. If using a conventional linear accelerator, the patient will therefore need to rotate with respect to the treatment beam to replicate contemporary treatments. Some specifically designed chairs for upright patients have an inbuilt rotating platform suitable for this purpose, but an independent mechanism such as a turntable is also acceptable and could accommodate a range of supports (16). The platform or turntable should be able to rotate continuously or to prescribed angles with a high level of accuracy, and ideally achieve 360° of rotation (17). If the turntable is not permanently in place in the treatment suite, it must be reproducibly positioned each time and calibrated to ensure alignment of its centre of rotation with the imaging and treatment beam isocentre (18). The patient support should feature independent translational adjustment of the patient position relative to the turntable, to facilitate alignment of the target to the rotational and treatment beam isocentres.

When treating a patient in an upright position, a challenge is introduced due to the potential loss of the stability typically provided by the treatment couch for recumbent patients. Patient immobilization devices connected to a turntable, which can rotate the patient relative to the static imaging and treatment beams. The level of support required, such as a seat, stool, or standing frame, will depend on the target location, performance status of the patient, and their ability to remain in the required position for the duration of the procedure. Additional fixation devices, analogous to those currently used in conjunction with the usual couch, can be adapted to be compatible with upright supports mounted on the rotating system (19). Several examples of in-house upright patient positioning have been reported. To treat a thymic carcinoma, a standing patient was secured to a back support with the aid of a belt (20). A similar approach could be used for prostate, rectal or gynaecological patients. A method has been evaluated for treating head and neck cancer (HNC) patients with a forward tilting chair. A thermoplastic mould was wrapped around the back of the head, connecting to a plate with a variable angle. This method was reported to have adequate reproducibility and patient comfort making it a viable option for positioning HNC patients upright (21). Based on an *ad hoc* method of breast immobilisation for upright treatment, thermoplastic moulds could be used for breast shape optimisation (22). Due to the potential reduction in respiratory motion for upright patients, the need for motion management such as free-breathing gating or breath-hold for thoracic and upper abdominal tumours may be decreased (8). Recently, systems for upright patient positioning have been developed, including a chair designed to position and rotate patients for HNC treatment using fixed beamline carbon ion therapy (16, 23). Patient stability may be aided by novel solutions, such as soft robotics, as currently used in rehabilitation (24). Several implementations of soft robotics currently being assessed include mask free HNC treatment, upright patient stabilisation and patient stability for horizontal rotation (25–27). The HNC example uses a camera to monitor head position and a pneumatic air bladder system to control position. A similar approach could be adapted for upright patients. While not commercially available yet, this supports the potential for upright patient positioning to be clinically implemented in the near future.

## Simulation Imaging

Simulation images should be a 3D representation of the anatomy in the treatment position. Anatomical changes between supine and upright images may be significant in the thoracic, abdominal, and pelvic regions due to gravity and posture. In some cases, these changes are the motivation for pursuing upright orientation. Image quality must be adequate to accurately delineate the target volumes and nearby critical organs and be free from geometric distortion. Voxel data must be converted into media information for dose calculation, which typically relies on accurate Hounsfield Units (HU) in the image or an accurate method of mapping material compositions and densities to an acquired image. Standard CT scanners are unable to image an upright patient, making an alternative imaging approach necessary. Dedicated vertical CT scanners exist which can produce the required images (20). However, these are highly specialised, and access to such a system is likely to be limited for most clinics.

A method has been proposed that uses the onboard imaging capabilities of linacs, removing the need for specialist equipment (28). The kV source and detector are fixed and projections are acquired as a seated patient is rotated at the isocentre, producing a cone-beam computed tomography (CBCT) scan. While the original approach used the treatment couch to perform the rotation, it could be substituted for a turntable, permitting a greater variety of patient positioning and an improved imaging speed. 4DCT is also an important part of simulation imaging for tumours subject to respiratory motion (29). Respiratory trace acquisition can be achieved similarly in the upright position using existing respiratory belts or through the use of projection data to derive the respiratory signal (30, 31).

The large radiation angle and detector required in CBCT increases scattered radiation reaching the detector, decreasing image contrast and rendering the HU inaccurate (32). To use CBCT images for treatment planning, improvements in image quality and HU consistency are required. Methods to estimate and correct for the influence of scatter on CBCTs include analytical and Monte Carlo based approaches, and more recently neural network and use of the linear Boltzmann equation (33–39). Other methods for improving CT number accuracy for dose calculations without scatter estimations include creating a synthetic CT *via* a bulk density override, generative adversarial network (GAN) or deformable image registration (DIR) (40–43).

The use of DIR may also be crucial for fusing multiple imaging modalities to deliver accurate radiation therapy to upright patients (44, 45). Intermodality fusion is already a standard part of supine treatment, used to assist with target and critical organ delineation (46). However, image fusion normally occurs between two images acquired with the same patient orientation. The larger anatomical differences between supine imaging modalities (like MR and PET imaging) and upright images may limit the accuracy of directly applying DIR. The upright case may require alternative strategies such as an intermediate step: first implementing the standard supine image fusion with diagnostic images to create required contours. DIR from supine CT to upright CBCT could transfer contours. Research has been undertaken to correct for large anatomical changes and lung changes in DIR, which has the potential to benefit supine-to-upright deformation (47, 48). The issues of image handling, registration and fusion may be the area requiring the most attention to realise routine upright radiotherapy.

## Treatment Planning

The treatment planning process involves the determination of the appropriate beam angles or arc delivery angles, followed by optimisation of the beam apertures and fluences to achieve the planning goals (49). To deliver upright radiotherapy using standard linacs, gantry rotation would be transposed to patient rotation. Mechanical limitations including rotation speed, acceleration and angular range would need to be constrained in forward and inverse planning. A further consideration may be required on speed, acceleration and jerk with respect to patient comfort: this may be used as a constraint/objective in inverse planning of arc treatments. Speed and acceleration tolerances may vary between patients, making this a variable constraint to be considered to ensure patient comfort.

As with supine treatment, the requirements of treatment planning depend on the treatment to be delivered. Simpler treatments, such as 3D conformal radiation therapy (3DCRT) and intensity modulated radiotherapy (IMRT), require multiple beams at different angles around the patient. Typically a multileaf collimator (MLC) is used to shape the beam to the tumour for each angle or modulate the beam intensity (50, 51). Provided the upright patient DICOM simulation images can be imported into the software, current commercial treatment planning systems (TPS) can achieve this through maintenance of a static gantry angle and the use of couch angles to achieve the different beam angles. Treatment planning complexity increases with continuously moving beams such as in dynamic conformal arc therapy (DCAT) and Volumetric Modulated Arc Therapy (VMAT). A feedback loop will be necessary to ensure turntable rotation accuracy, especially for VMAT treatment with variable rotation speed. With minor modifications to optimisation, in which the beam angle is replaced by a turntable/couch angle, both DCAT and VMAT treatments could be planned with little difference compared with supine/prone treatment position.

Non-coplanar treatment to an upright patient with a conventional medical linac may also be feasible. Instead of using only a fixed gantry angle, it could be altered within a small range (likely dependent on immobilization). Further non-coplanar beam angle range could be achieved through the tilt of the patient positioning device. Most current TPS do not accept images in the upright patient geometry, with the exception of some particle therapy systems – the existence of which demonstrates that it is already implementable (52). With vendor cooperation, TPS capabilities could be extended to accept upright images. The ability of TPS to perform related tasks, such as image fusion and dose accumulation, with upright images would also be advantageous.

## Online Setup and Image Guidance

Patient positioning for treatment as per simulation typically involves set up using the external patient contour followed by verification using imaging of the target and internal anatomy. Initial patient setup to the machine isocentre is typically achieved using lasers aligned to tattoos on the patient skin, or through surface guidance. In the upright scenario, a ‘reference’ position/rotation must be defined, which is how the patient will be set up before treatment. This may be the position of the first static treatment beam or the start of the first arc. Lasers and surface tracking may still be applied in the upright position, with optical or thermal surface guidance providing an elegant solution for upright patient positioning (53).

Once the patient is set up in the treatment position, images are acquired to validate anatomy and tumour position before treatment (54). Positional adjustments may then be required, potentially involving manually repositioning the patient or shifting the position of the treatment chair if it allows for 6 degrees of freedom (like current treatment couches). These images can be planar x-ray images or 3D volumetric images. While the image quality does not need to be the same as the simulation image, it still needs to be sufficient to validate patient alignment to ensure that the tumour receives the intended dose (54). Thus, the imaging system on a standard linac can be used to image an upright patient and allows for 2D and/or 3D setup image acquisition before treatment. The acquisition of simulation images using the same system as treatment may facilitate online adaptive radiotherapy, to account for anatomical changes or variations in daily patient positioning (55, 56).

Continuous monitoring of the patient’s position during treatment could be achieved by using surface guidance to track the patient’s external contour, with reference position updated for each beam (57). This will be more challenging for treatment with continuous rotation (like VMAT), requiring constant updating of the reference image. Positional monitoring could also be achieved *via* images acquired during treatment. If the imaging geometry is such that imaging and treatment can be performed contemporaneously then intra-treatment planar imaging could be used to track internal target anatomy during patient rotation using systems such as beacons or radio-opaque markers (58).

## Discussion

Delivering upright radiation therapy to an upright patient is achievable for the most part with current technology. Engagement from vendors would be required to make the treatment planning more clinically acceptable. Vendor cooperation could also aid in the production of simulation images through a formalised acquisition and correction pipeline. While not previously discussed, the shielding requirements for upright radiation therapy must be considered prior to the commencement of treatment. Assuming a fixed horizontal treatment beam for upright radiation therapy, there will only be one primary barrier. Depending on the department, this barrier may not have the required shielding and must be increased, adding an additional requirement for upright radiation therapy. However, concentrating the radiation, and thus the shielding, in one direction would reduce the total amount of shielding required (59). This reduction, coupled with the potential decrease in cost and size of a fixed beam linac could lead to the potential for portable radiation therapy. It has been proposed to contain the equipment needed for radiation therapy in a truck or shipping container (59).

The framework paper is focused on high-quality MV photon treatment with the same geometric accuracy as recumbent treatment. However, the implementation of such treatment could serve as a gateway to particle therapy. Particle therapies produce advantageous dose distributions resulting in less damage to the normal tissue. However, delivering particle therapy to patients in conventional recumbent positions requires large rotating gantries which come at a considerable cost and technical complexity (60). Particle therapy facilities are frequently constructed with fixed beamlines, even when one or more gantries are included. Advances in patient positioning and rotation in front of a fixed beamline aperture may improve the usefulness of those fixed beams. Treatment of a patient in an upright position may also help to facilitate the use of Synchrotron radiation for emerging techniques such as microbeam radiation therapy (MRT) and FLASH radiotherapy (61, 62). Creating a practical workflow for upright radiation therapy on clinical linacs should help to progress research into upright particle and FLASH radiation therapy aiding their progression towards clinical implementation.

## Conclusion

While radiation therapy is traditionally delivered to recumbent patients, we have provided a framework to deliver contemporary highly modulated radiation therapy to upright patients. Requirements that must be met for the four aspects of radiation therapy – patient positioning, simulation imaging, treatment planning and setup and image guidance – have been considered. We have identified the most promising developments in each of these areas that could lead to viable solutions, such that implementation in the near future is realistic and feasible. The framework has been written with the intent of routinely delivering upright radiation therapy in current centres with existing facilities and minimal new equipment. However, elements of the framework could be applied to other contexts where upright radiation therapy is preferable. It is hoped that introducing upright radiation therapy could allow patients to be more comfortable during their treatment, receive less normal tissue irradiation or have greater access to treatment.

## Data Availability Statement

The original contributions presented in the study are included in the article/supplementary material. Further inquiries can be directed to the corresponding author.

## Author Contributions

All authors listed have made a substantial, direct, and intellectual contribution to the work, and approved it for publication.

## Conflict of Interest

NH and TK receive collaborative research funding from Varian Medical Systems and Reflexion Medical for unrelated projects.

The remaining authors declare that the research was conducted in the absence of any commercial or financial relationships that could be construed as a potential conflict of interest.

## Publisher’s Note

All claims expressed in this article are solely those of the authors and do not necessarily represent those of their affiliated organizations, or those of the publisher, the editors and the reviewers. Any product that may be evaluated in this article, or claim that may be made by its manufacturer, is not guaranteed or endorsed by the publisher.
